# Corrective Breast Surgery in Adolescents and Young Adults: A Retrospective Patient-Reported Outcome Study Using the BREAST-Q Questionnaire

**DOI:** 10.1007/s00266-026-05859-3

**Published:** 2026-06-02

**Authors:** Riham Kheir, Ehud Fliss, Dana Adelson, Sapir Davidov, Eyal Gur, Yoav Barnea, Gal Bracha, Ehud Arad

**Affiliations:** 1 Department of Plastic and Reconstructive Surgery, Tel Aviv Sourasky Medical Center, 6 Weizman Street, 64239 Tel-Aviv, Israel; 2 Affiliated to the Sackler School of Medicine, Tel Aviv University, Tel Aviv, Israel

**Keywords:** BREAST-Q, Adolescent breast surgery, Breast corrective surgery

## Abstract

**Background:**

Breast corrective surgery has been on the rise in the adolescent population in recent years. Data regarding the effects of surgery on quality of life (QOL) and satisfaction in this patient population are required for accurate risk–benefit evaluation. The objective of this study was to assess the satisfaction and effect of breast corrective surgery on QOL in the adolescent and young adult population, using the validated BREAST-Q questionnaire.

**Methods:**

Included were all patients aged 21 and under, who underwent breast corrective surgery due to macromastia, breast asymmetry and gynecomastia during the years 2013–2021. Pre- and postoperative BREAST-Q questionnaires were retrospectively completed. QOL and satisfaction measures were assessed and compared before and after surgery.

**Results:**

Fifty-two patients were included, of whom 24 (46.2%) underwent asymmetry correction, 18 (34.6%) underwent reduction mammoplasty and 10 (19.2%) underwent gynecomastia repair. A significant improvement in psychosocial well-being, sexual well-being and satisfaction with the breasts was recorded following surgery in all groups. Physical well-being scores were improved for patients following breast reduction and gynecomastia repair only. Within the breast asymmetry group, correction with implants was associated with decreased physical well-being, while correction without implants was associated with improved physical well-being and the difference between these two subgroups was statistically significant.

**Conclusions:**

The findings of this study suggest that in adolescents and young adults, breast corrective surgery including reduction mammoplasty, asymmetry correction and gynecomastia repair is associated with high satisfaction scores and improved QOL according to the BREAST-Q questionnaire.

**Level of Evidence III:**

This journal requires that authors assign a level of evidence to each article. For a full description of these evidence-based medicine ratings, please refer to the Table of Contents or the online Instructions to Authors www.springer.com/00266.

## Takeaway Points

- Breast deformities have been shown to negatively affect physical and psychosocial measures in the adult population.
- Adolescent and young adults may face various breast deformities (e.g. macromastia, breast asymmetry and gynecomastia) that may interfere with physical and social functioning.
- Corrective breast surgery in the adolescent and young adult population is associated with high satisfaction scores and improved QOL measures, as demonstrated in the BREAST-Q questionnaire.

## Introduction

Breast corrective surgery in adolescents accounts for approximately 4% of all cosmetic breast surgery in the USA and is on the rise [1]. The most common procedures include reduction mammaplasty, breast augmentation and gynecomastia repair. The negative physical and psychological impact of macromastia, breast asymmetry and gynecomastia has been well documented in the adult population [2–7]. The dramatic improvement in self-esteem and quality of life (QOL) has also been described in adults, based on validated patient-reported outcomes studies (PROM) [8–13]. Nevertheless, very little has been published regarding the benefits of breast corrective surgery in adolescents and young adults.

The adolescent population is distinct from the pediatric or adult populations both in physiological and psychosocial parameters and thus deserves separate focus. Studies demonstrate the generic benefit and safety of plastic surgery in adolescents [14–18]. Corrective surgery that is performed at an early stage of development can enhance the ability for the patient to develop self-confidence and have an overall positive impact on the patient’s future. Breast surgery in particular may promote a positive psychosocial environmental perception [19–22]. Nevertheless, it is especially important to justify the benefit of surgical intervention at this early stage, perhaps before physical and emotional maturity has been reached [23, 24]. Physicians must weigh the benefits of alleviating physical and psychosocial difficulties at an earlier stage (aspiring to prevent permanent developmental damage), against potential drawbacks such as physical relapse of deformity with further growth, or immaturity in expectations and in coping with the surgical process and outcomes. For this end, it is of outmost importance to focus on the subjective perspectives of patients regarding the benefits of early surgery, in order to prove the value of early intervention on the patient’s outcome.

Measurement of PROM has become increasingly important in cosmetic and reconstructive breast surgery. The BREAST-Q questionnaire is a validated breast surgery-specific PROM tool, measuring satisfaction with surgery, QOL and patient experience, and has emerged as a popular tool in the evaluation of PROM and general well-being assessment [24–26]. Few studies have incorporated the BREAST-Q in research of the adolescent population [27, 28]. Our group has initiated a prospective study of this topic, but also conducted a preliminary retrospective study, the results of which are presented here.

## Methods

This is a retrospective study and is preliminary to a similar prospective cohort study, which we initiated in 2021. Both studies were approved by our institutional research ethics board. The present study includes all patients aged 21 years or younger, who underwent breast corrective surgery in the years 2013–2021. The 21-year age cutoff was selected in accordance with prior adolescent plastic surgery literature [28–31], which often defines adolescence more broadly to encompass individuals from puberty through early adulthood (up to 21 years) to reflect ongoing psychosocial and developmental considerations. (This will be further discussed.) Furthermore, for each group we performed a sub-analysis of outcomes specifically for the younger patients (up to the age of 19), to verify whether the trends were similar to those of the entire group.

All surgeries were performed by the senior author. Male and female patients were included and divided into three groups: reduction mammaplasty, breast asymmetry correction, and gynecomastia repair. Some female patients presented with both breast hypertrophy and asymmetry, potentially applicable for both groups of breast reduction or asymmetry repair. Allocation to study groups for these patients was done according to the following criteria: If both breasts were reduced by 300g of tissue or more, the patient was included in the reduction group; if one or both breasts were reduced by less than 300g (considered mastopexy), the patient was included in the asymmetry group [32]. The rationale for this is that breast reduction patients present with more physical symptoms (e.g., back aches), compared to asymmetry patients that present with more prominent psychosocial difficulties.

### Surgical Techniques

All reduction mammaplasties were performed using a superomedial pedicle and a wise pattern skin resection technique. In asymmetry patients, the mastopexy side was performed using a wise pattern with either a superior or superomedial pedicle. Augmentations were performed through an inframammary incision, while the plane of the implant was selected based on pinch test cutoff of 2 cm and the quality of skin envelope. (For example, tuberous breasts requiring envelope expansion usually received subglandular implants.) Regarding the gynecomastia group, the surgical technique was chosen in correspondence with Simon classification [33]. All cases had liposuction with or without open gland resection through a subareolar or periareolar incision, depending on skin and breast tissue redundancy and type.

Demographic and clinical data were collected from patients’ charts, including age, medical history, smoking, procedure details, complications, and the number of interventions. Patients were contacted and offered to participate in the study by completing an appropriate BREAST-Q questionnaire. Following a thorough explanation provided by our surveyors, patients were asked to retrospectively fill out BREAST-Q surveys, pertaining to their preoperative and postoperative phases, separately. All female patients were surveyed by a single female medical student and all male patients by a single male resident, in order to increase consistency and minimize variance in subjective data collection.

BREAST-Q was used under license from Memorial Sloan Kettering Cancer Center and the University of British Columbia. All patients signed consent forms in order to participate in the study; patients younger than 18 signed an appropriate consent form along with at least one of their parents.

### BREAST-Q Questionnaire

We used 2 procedure-specific modules of the BREAST-Q—the augmentation and the reduction/mastopexy modules. The augmentation module was completed by patients who had received breast implants for asymmetry correction. The reduction/mastopexy module was completed by patients who had undergone reduction mammaplasty, mastopexy or gynecomastia repair. Patients, who had undergone asymmetry correction by both reduction/mastopexy and insertion of implants, completed both modules. For this subgroup, statistical analysis was based on the augmentation model only since this was the prominent feature of their correction. Additional items were extracted from the reduction/mastopexy module in order to take into account specific data that do not exist in the augmentation module (scar-related items). Although each module addresses procedure-specific outcomes, score interpretation is inherently complex and comparisons across surgical groups must be interpreted with caution.

### Statistical Analysis

The BREAST-Q score for each patient was calculated using the Q-score program, which converts raw survey scores of 1 through 4 or 5 to continuous scores of 0 to 100. Scores were entered into a secure database according to patients and employed modules.

The database was analyzed by SPSS statistical package (Version 28, IBM; Armonk, NY). Descriptive statistics was used to describe the distribution of variables associated with characteristics of the study sample. Continuous variables were presented as means ± standard deviation, median and interquartile range (IQR) and dichotomous/categorical variables as proportions. To test differences in independent continuous variables between two or more groups, nonparametric tests such as Mann–Whitney and Kruskal–Wallis were performed, respectively. Wilcoxon test was used to compare between two paired groups.

## Results

Fifty-six patients were offered to participate in this study, of whom 52 (92.9%) agreed and 6 (7.1%) either declined or were unavailable. No systematic, demographic or clinical differences were identified between respondents and non-respondents based on the chart review. Of the 52 respondents, 24 (46.2%) underwent asymmetry correction, 18 (34.6%) underwent bilateral breast reduction surgery, and 10 (19.2%) underwent gynecomastia repair. Of the 24 asymmetry patients, 16 (66.7%) underwent combined augmentation–mastopexy, 6 (25%) underwent reduction/mastopexy only and 2 (8.3%) underwent augmentation only. Of the 18 asymmetry patients that received implants, 15 (83.3%) had implants placed in a subglandular or subfascial plane, and 3 (16.7%) in a dual subpectoral plane.

**Fig. 1:**
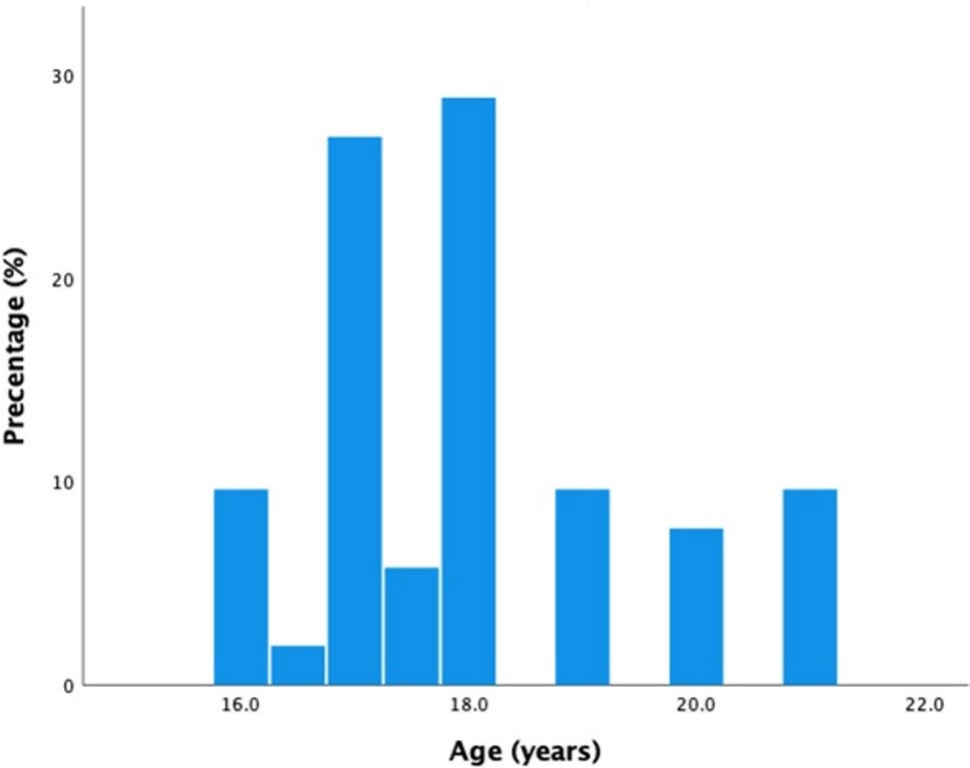
Age distribution of study population

Mean age of the entire cohort was 18 years (range 16–21), with the majority of patients being between the age of 16 and 19 (*n* =43, 82.7%) (Table 1, Fig. 1). Mean body mass index (BMI) was 25. Three patients (5.8%) were smokers, who reportedly ceased smoking 1 month prior to surgery, in Table 2.

**Table 1:** Age distribution of the study cohort

Age (years)	Frequency (*N*)	Percent (%)	Cumulative percent (%)
16.0	5	9.6	9.6
16.5	1	1.9	11.5
17.0	14	26.9	38.5
17.5	3	5.8	44.2
18.0	15	28.8	73.1
19.0	5	9.6	82.7
20.0	4	7.7	90.4
21.0	5	9.6	100.0
Total	52	100.0

**Table 2:** Descriptive demographics

	Surgery						*p* value
	Total *N*=52	Reduction *N*=18	Asymmetry *N*=24			Gynecomastia *N*=10	
			Augmentation *N*=2	Augmentation–Mastopexy *N*=16	Mastopexy *N*=6		
Age, years (mean ± SD)	18 ± 1.4	17.7 ± 1.3	19 ± 2.8	18.4 ± 1.35	17.8 ± 1.6	17.8 ± 1.4	0.277
Follow-up, months (mean, range)	31.3 ± 24.6	34.6 ± 27.5	44 ± 14.1	29.4 ±21.9	30.2 ± 39.5	26.6 ± 14.4	0.536
BMI (mean ± SD)	24.6 ± 3.6	26.4 ± 3.1	18.5 ± 5.6	23.5 ± 3.2	23.3 ± 3	25 ± 3.4	0.009
Smoking, *N* (%)	3 (5.8)	2 (11.1)	0 (0)	0 (0)	0 (0)	1 (10)	0.355
Comorbidities, *N* (%)	5 (9.6)	2 (11.1)	2 (100)	0 (0)	1 (16.67)	0 (0)	0.102
Asthma, *N* (%)	2 (3.8)	2(11.1)	0 (0)	0 (0)	0 (0)	0 (0)	N/A
Hypothyroidism, *N* (%)	1 (1.9)	0 (0)	1 (50)	0 (0)	0 (0)	0 (0)	N/A
Poland syndrome, *N* (%)	1 (1.9)	0 (0)	1 (50)	0 (0)	0 (0)	0 (0)	N/A
Liver transplant, *N* (%)	1 (1.9)	0 (0)	0 (0)	0 (0)	1 (16.67)	0 (0)	N/A

*BMI* body mass index; *SD* standard deviation; *N/A* non-applicable

There were five patients (9%) with notable minor complications: two cases of seroma and delayed healing in the asymmetry group; two cases of dehiscence; and one case of partial areolar necrosis in the reduction group (all healed secondarily). There were three reoperations, all from the asymmetry group. (Two underwent periareolar re-tightening, and one underwent replacement of implant for size.) Mean follow-up for the entire cohort was 31.3 months (range 4–108).

### Asymmetry Corrective Surgery (*N* = 24)

**Fig. 2:**
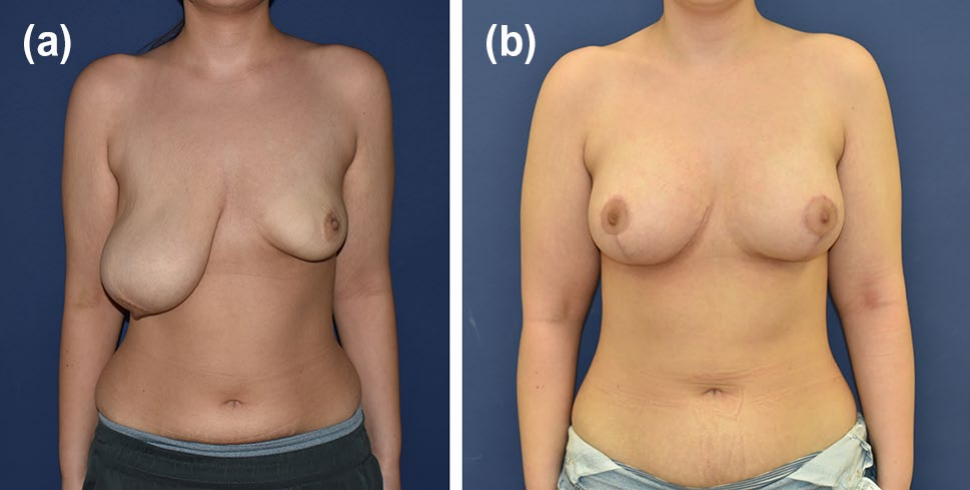
**a** Preoperative photograph of a 16-year-old female patient, with breast asymmetry. **b** The patient two years after undergoing asymmetry correction, with right breast reduction augmentation, and left mastopexy augmentation

Mean age was 18.3 years, average BMI was 23, and there were no active smokers. This group demonstrated a statistically significant improvement in psychosocial well-being, satisfaction with the breast and sexual well-being following surgery (*p* value < 0.001), in Table 3. In contrast, a non-statistically significant *decrease* in physical well-being was noted. The median score of satisfaction with the nipple was 17 (out of 20), satisfaction score with implants (when applicable) was 8 (out of 8), and overall satisfaction with outcome was 100 (out of 100). In addition, we examined the satisfaction results in the same cohort of patients, examining specifically patients up to the age of 19 years old (*N* = 14). The results for this cohort were similar, demonstrating statistically significant postoperative improvement in psychosocial well-being, sexual well-being, and satisfaction with breast (*p* = 0.001) (Fig. 2).

**Table 3:** Pre- and postoperative BREAST-Q according to surgical procedure

		Reduction Total = 18			Asymmetry Total =24			Gynecomastia Total=10			*p* value
	Score	Median (IQR)	Range	95% CI, *p* value	Median (IQR)	Range	95% CI, *p* value	Median (IQR)	Range	95% CI, *p* value	
Psychosocial well-being	Pre-op	33 (22.25–72)	14–66	**(40–56.5), <0.001**	38.5 (25–49)	0–68	**(28.–58.5), <0.001**	35 (24–74)	14-50	**(58-82), 0.005**	0.713
	Post-op	81 (72–98.25)	52–100		89.5 (69.5–100)	39–100		100 (100–100)	69-100		**0.013**^**a,e**^
Physical well-being	Pre-op	40 (34–47)	6–51	**(31–47.9), <0.001**	100 (87–100)	40–100	**(–9–0.00) 0.410**	82 (72–100)	72-100	**(0-28), 0.042**	<0.001*^d^
	Post-op	77 (65–90)	54–100		91 (74.25–100)	52–100		100 (90–100)	77-100		0.030**^d^
Sexual well-being	Pre-op	38 (31.75–46.25)	0–90	**(21.5–55.5),** **<0.001**	33 (13–44)	0–58	**(42–67), <0.001**	34 (0–43.75)	0-65	**(53-100), 0.011**	0.399
	Post-op	79 (65–100)	44–100		84 (67–100)	39–100		100 (100–100)	100-100		**0.017**^**b,f**^
Satisfaction with breast	Pre-op	27.5 (21–35)	0–53	**(49–65), <0.001**	28 (18–40.25)	0–63	**(39–63), <0.001**	21 (11–36)	0-52	**(48-89), 0.005**	0.52
	Post-op	82 (73–90.5)	63–100		80 (71–91)	40–100		100 (86–100)	66-100		**0.036**^**c,g**^
	Satisfaction with Outcome	100 (86–100)	76–100	N/A	100 (75.25–100)	41–100	N/A	100 (100–100)	86-100	N/A	0.074
	Satisfaction with implants (post-op)§	N/A	N/A	N/A	8 (8–8)	6–8	N/A	N/A	N/A	N/A	**N/A**
	Satisfaction with nipples (Post-op)	18.5 (16–19.75)	9–20	N/A	17 (14–18) ɸ	8–20	N/A	19 (18–20)	15-20	N/A	0.196
	Satisfaction with office staff (Post-op)	100 (100–100)	41–100	N/A	100 (100–100)	55–100	N/A	100 (100–100)	100-100	N/A	0.333
	Satisfaction with medical team (Post-op)	100 (86–100)	0–100	N/A	100 (95.5–100)	0–100	N/A	100 (100–100)	100-100	N/A	0.119
	Satisfaction with surgeon (Post-op)	100 (100–100)	81–100	N/A	100 (94.75–100)	65–100	N/A	100 (100–100)	100-100	N/A	0.231
	Satisfaction with information (Post-op)	95 (79–100)	60–100	N/A	84 (66.5–85)	51–100	N/A	100 (90–100)	57-100	N/A	0.003****^,j^

*Post-op* postoperative; *Pre-op* preoperative; *N/A* non-applicable, *IQR* interquartile range, *CI* confidence interval

Values in bold represent statistical significance

^§^Only for augmentation score, *N* = 17 (1 missing)

ɸ Only for mastopexy, *N* = 21

Comparison between reduction and asymmetry –

*Physical well-being score preoperative is significantly different with *p* value<0.001

****Satisfaction with information is significantly different with *p* value= 0.036

Comparison between reduction and gynecomastia –

^a^Psychosocial well-being score postoperative is significantly different with *p* value 0.004

^b^Sexual well-being score postoperative is significantly different with *p* value 0.013

^c^Satisfaction with breast score is significantly different with *p* value 0.027

^d^Physical well-being score pre- and postoperative is significantly different with *p* value<0.001 and 0.014, respectively.

Comparison between asymmetry and gynecomastia –

^e^ Psychosocial well-being score postoperative is significantly different with *p* value 0.01

^f^ Sexual well-being score postoperative is significantly different with *p* value 0.013

^g^ Satisfaction with breast post-op score is significantly different with *p* value 0.002

^j^ Satisfaction with information is significantly different with *p* value= 0.002

In order to further explore the decrease in physical well-being, a second analysis was performed by comparing the physical well-being of asymmetry patients who underwent correction with implants to those without implants (Table 4). The change in physical well-being following surgery demonstrated a statistically significant difference as patients treated with implants had a median *decrease* of 4.5 points in physical well-being score, while patients treated without implants had a median *increase* of 8 points (*p* *=* 0.014). This significant difference was mainly attributed to lower postoperative scores in both items of “breast pain” and “breast tension” within the physical well-being domain, in asymmetry patients with implants (*p* = 0.08).

**Table 4:** Comparison between physical well-being score within asymmetry group

	Group	*N* ɸ	Median score (IQR)	*p* value
Preoperative physical well-being	With implant	18	100 (96.75–100)	0.081
	No implant	6	78 (52–100)	
Postoperative physical well-being	With implant	18	91 (73–100)	0.266
	No implant	6	100 (85.5–100)	
Change in physical well-being score following surgery	With implant	18	− 4.5 (−18.75–0)	**0.014**
	No implant	6	8 (0–40.5)	

ɸ N indicates the number of questionnaires completed

Values in bold represent statistical significance

### Reduction Mammoplasty (*N* = 18)

**Fig. 3:**
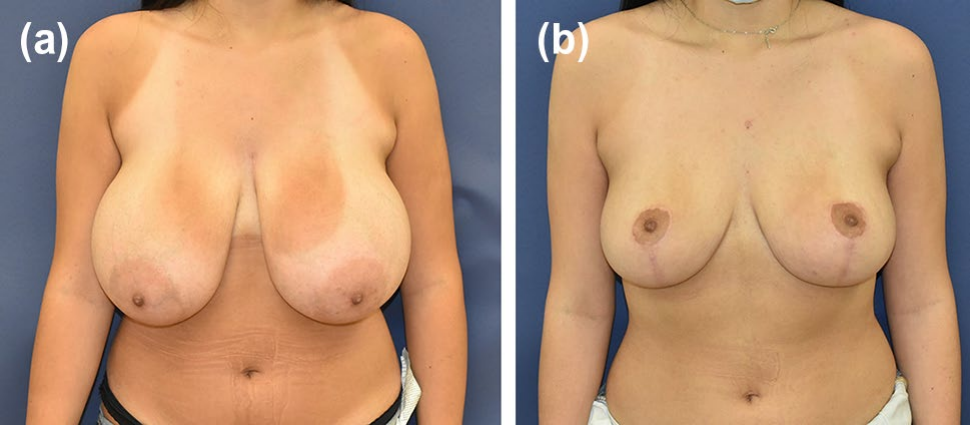
**a** Preoperative photograph of a 17-year-old female patient, with breast hypertrophy. **b** The patient 12 months after undergoing reduction mammaplasty, with superomedial pedicle and wise pattern skin resection

Patients’ mean age was 17.7, mean BMI was 26.4, and two patients (11.1%) were active smokers. This group of patients demonstrated a statistically significant improvement in all of the BREAST-Q subsets following surgery (*p* value<0.001), in Table 3. Overall satisfaction with outcome median score 100 (86–100 IQR) and satisfaction with the nipples was 18.5 (out of 20). Similarly, satisfaction results of patients up to the age of 19 years old (*N* = 15) demonstrated significant postoperative improvement in all aspects—psychosocial well-being, physical well-being, sexual well-being, and satisfaction with breast (*p*= 0.001), in Fig. 3.

### Gynecomastia Corrective Surgery (*N* = 10)

**Fig. 4:**
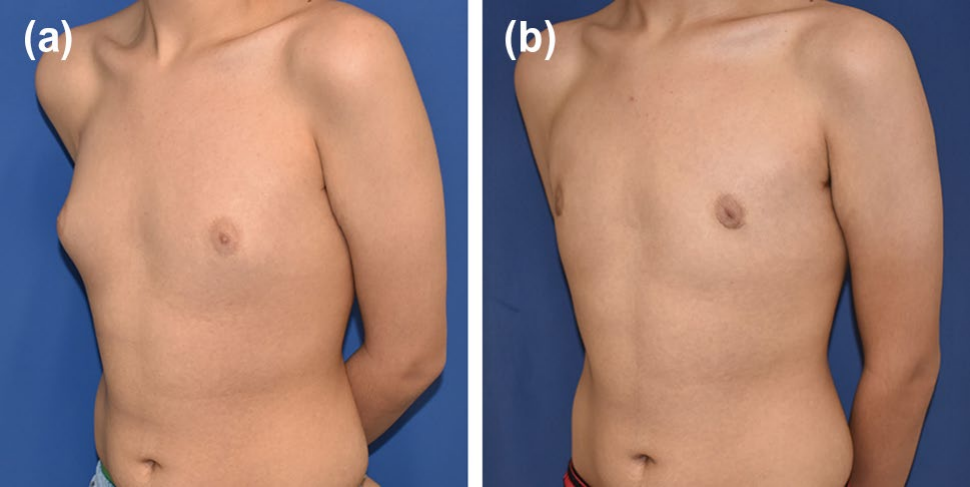
**a** Preoperative photograph of a 16-year-old male patient, with idiopathic gynecomastia. **b** The patient 9 months after undergoing resection and liposuction of bilateral gynecomastia with a periareolar incision

Patients’ mean age was 18, mean BMI was 25, and one patient was an active smoker (10%). This group demonstrated a statistically significant improvement in all BREAST-Q subsets following surgery (psychosocial well-being, physical well-being, sexual well-being and satisfaction with breast), (*p* value = 0.005, 0.042, 0.011 and 0.005, respectively), in Table 3. Overall satisfaction with outcome median score was 100 (100–100 IQR). Satisfaction with the nipple was 19.5 (out of 20). This was true for patients up to the age of 19 years old (*N* = 9), showing a significant postoperative improvement in psychosocial well-being, physical well-being, sexual well-being and satisfaction with breast (*p* = 0.008, 0.042, 0.017, 0.008 respectively), in Fig. 4.

## Discussion

Developmental deformities of the breast may cause body image disorders and lead to depression, psychological distress and eating disorders in both adolescents and adults [34–38]. Corrective breast surgery has been shown to have a positive psychosocial impact, as manifested by improvement in PROM following surgery [8–13]. Although studies regarding this subject most commonly involved the adult population, the limited literature in adolescents suggests that corrective breast surgery improves psychosocial and physical strain, alongside appearance-related burdens [39–41]. Surgery in adolescents has also been shown to be safe with a low rate of complications [19].

Our cohort includes participants up to the age of 21. There is no uniform age-specific definition for the period of adolescence, and it may differ from one country to another [42–44]. Adolescence is determined by social and behavioral factors, in addition to the biological factors of physical growth and breast growth cessation. Thus, some recent studies suggest that rather than age 10-19 years, a broader definition from puberty to the mid-20s corresponds more closely to adolescent growth and popular understandings of this life phase [16, 45, 46]. Nevertheless, our cohort is still composed mainly of patients 19 and under (82.7%), and the results of the age-specific analysis comparing outcomes of patients aged 19 and, under to the broader group of patients, showed similarity in outcomes.

As younger patients are seeking surgical solutions to relieve severe emotional and physical hardships, surgeons and physicians are faced with age-related dilemmas. Specifically, the notion of performing breast corrective surgery at a young age raises three main concerns: (1) Will the breast continue to grow following surgery and require reoperation? (2) Are adolescents sufficiently mature and mentally composed to cope with surgery and be content with its outcome? (3) Will surgery contribute to significant improvement in patients’ well-being and quality of life?

Our present study is too limited in numbers and in follow-up to draw any conclusions regarding postoperative breast growth. Breast surgery in adolescents runs the theoretical risk of continued breast growth and development after surgery. Although continued breast growth may potentially alter postoperative results, this is most probably *not* age-specific but rather more closely related to patient-specific characteristics such as age at menarche and BMI. Breast growth probably reaches completion by 3 years following menarche, hence by the age of 14–16 [29, 30, 47, 48]. Current literature favors the notion that surgical intervention should be performed after a period of stabilization in breast shape and size and this does not necessarily correspond to an arbitrary age cutoff [47].

Our present study aims to provide preliminary data regarding both the coping experience of these young patients with the surgical process and the contribution of surgery to PROM of QOL—thus addressing the latter two questions above. The ability of any adolescent patient to cope with surgery, realistic surgical outcomes and possible postoperative complications must be thoroughly assessed prior to surgery. Early corrective surgery is not appropriate for everyone, distressed as one may be from the condition of one’s breast. This is addressed in our institution by a multi-disciplinary team consisting of a plastic surgeon, pediatrician, endocrinologist, adolescent psychologist and social worker, as this allows for a comprehensive assessment of the adolescent’s physical and mental readiness for such surgery. All patients that are included in this study have therefore been approved for surgery based on being fit by the criteria above.

Choice of surgical technique was based on patients’ physical and psychological complaints and expectations. It is extremely important to explore with the patients and their parents all possible strategies with their respective trade-offs, perhaps more elaborately than necessary in the adult patients. The results of this study demonstrate a very high satisfaction rate with the surgical team and with information provided, validating that these young patients, who were approved for surgery and guided through the preparatory phases of decision-making, were in fact “on board” with the process and the selected surgical plan.

The main goal of our study was to collect evidence-based data regarding the impact of surgery on the various aspects of patient QOL. To that extent, the BREAST-Q questionnaire is a valuable validated tool [21, 22]. It should be noted, however, that the BREAST-Q is not specifically intended to assess QOL in patients undergoing gynecomastia repair; however, for the purposes of this study we found it a valuable tool. In all three study groups, psychosocial well-being and sexual well-being were significantly improved after surgery (Table 3). In regard to physical well-being, a significant improvement was seen in the reduction and gynecomastia groups, however, not for the asymmetry correction group. Close examination of these results suggests that this was attributed mainly to patients who received implants. Interestingly, the presence of implants somehow had a negative impact on their physical well-being, apparently by causing tension and discomfort in physical activity. Unfortunately, we lack detailed information as to the possible symptoms that may have hampered physical well-being, but plan to address this in detail in our prospective study. Asymmetry patients who were treated with implants also had lower scores for scar location and appearance in comparison with those treated without implants, and this may also reflect the trend seen in physical well-being following surgery. These findings reiterate the importance of patient education regarding possible adverse symptoms associated with implants and remind surgeons that the decision to use implants in adolescents should not be taken lightly.

Within the satisfaction criteria of the BREAST-Q, we found a significant improvement in patients’ satisfaction with the breast after surgery and high satisfaction with outcome scores (Table 3). Satisfaction with outcome was highest in the gynecomastia repair group (98.6 ± 4.3), followed by the reduction mammoplasty group (94.2 ± 8.9) and asymmetry correction group (87 ± 17.1). The asymmetry group also reported lower rates of satisfaction with information regarding surgery (*p* = 0.003) and with the nipples. These slightly lower scores may be explained by the fact that admittedly, it is easier to attain good symmetry in reduction and gynecomastia patients where similar procedures are performed on both breasts, than in difficult asymmetry cases (e.g., tuberous breasts, Poland syndrome, etc.) in which differential procedures are employed with residual asymmetry often remaining. Nevertheless, these findings highlight the special need this group may have for extra care and clarity in regard to surgical choices and preparation for surgery.

Our findings add to previous studies demonstrating the benefits of corrective breast surgery in adolescents. Nuzzi et al. have published a series of prospective studies aimed to assess various outcome measures of breast surgery in adolescents [29–31]. Throughout this series of studies, the authors evaluated both surgical outcomes measures and QOL outcomes (Short form 36 (SF-36), Rosenberg’s self-esteem scale (RSES) and Eating Attitudes-26). These studies demonstrated significant improvement in physical, psychosocial and QOL measures and provide valuable information in a large group of patients; however, they are not based on PROM BREAST-Q methodology and therefore may be deficient in authenticity of patients’ subjective reports. Davis et al. [49] reported 11 adolescent patients with a mean age of 16.3 years who underwent reduction mammoplasty and completed a pre- and postoperative BREAST-Q questionnaire. Statistically significant improvement was seen in all categories of the questionnaire except sexual well-being. The highest predictor of overall patient satisfaction in the adolescent cohort was satisfaction with information (r=0.4859), reiterating the special need for clear communication with young adolescent patients. Our current study further supports the findings of these previous studies and adds validity due to the use of the BREAST-Q questionnaire.

Our study has several limitations. The small size and heterogeneous nature of our cohort retracts from its statistical power; while the findings are clinically relevant, they should be regarded as preliminary. Moreover, this is a retrospective study with inherent limitations. Preoperative patient-reported outcome data were retrieved retrospectively and are subjected to recall bias as the accuracy of the patients’ response regarding their preoperative state may have been biased to some degree by issues with recollection and/or by their current postoperative state. Since patients’ recollection of their preoperative state may be influenced by their postoperative experiences, there is a potential of overestimating or underestimating perceived benefits. This recall bias could affect the accuracy of paired comparisons, and results should be interpreted with caution. BREAST-Q scores may have considerable variance depending on comprehension and cooperation of participants. Additionally, as mentioned above, the BREAST-Q questionnaire is not validated for patients undergoing gynecomastia correction and this should be taken into account when interpreting the results. Last, the study population is highly heterogeneous and in fact includes several subgroups that were of medium size. Our ongoing prospective study is designed to address these limitations by collecting pre- and postoperative BREAST-Q data prospectively, enrolling a larger and more homogeneous cohort, and extending follow-up to better assess long-term outcomes and late complications.

## Conclusions

The findings of this study strengthen the notion that breast corrective surgery in adolescents and young adults is safe and associated with improved QOL and high satisfaction rates, as demonstrated in the BREAST-Q questionnaire. Although previous studies have examined adolescent and young adult breast surgery outcomes, few have utilized the BREAST-Q as a PROM measure. Our study adds to the growing literature while providing complementary data specific to this population. Larger prospective patient-reported outcome studies are required in order to continue and characterize the adolescent subpopulation of patients and improve our ability to tailor treatment to the expectations and needs of these patients.
